# Ileal free flap for hypopharynx reconstruction – case series

**DOI:** 10.1515/iss-2024-0005

**Published:** 2024-07-24

**Authors:** Luis Fernando Tintinago-Londoño, Estephania Candelo, Tania Guzmán, William Victoria-Morales

**Affiliations:** Departamento de Cirugía de Cabeza y Cuello, 655490 Fundación Valle del Lili, Cali, Valle del Cauca, Colombia; Centro de Investigaciones Clínicas, Fundación Valle del Lili, Cali, Valle del Cauca, Colombia; Facultad de Ciencias de la Salud, Universidad Icesi, Cali, Valle del Cauca, Colombia

**Keywords:** cervical esophagus, hypopharynx, ileal free flap, reconstructive surgery

## Abstract

**Objectives:**

Reconstructing the hypopharynx while preserving the larynx poses a complex surgical challenge due to the limited space and the high position of the hypopharynx in the neck. We present our experience with hypopharyngeal reconstruction and larynx preservation using an ileal free flap.

**Case presentation:**

Six consecutive cases were reported (age range 17–75; 2 females). Indications for surgery were tumor excision, postexcision flap failure, postradiation stenosis, caustic ingestion injury, and cervical esophageal perforation. The larynx was preserved in four cases. Graft survival rate was 100 %. Videofluoroscopic swallowing studies conducted at postoperative day 20–80 were normal in three cases. Two cases presented with stenosis but responded well to endoscopic dilations. Unfortunately, the third case expired due to tumor recurrence.

**Conclusions:**

The ileal free flap is a surgical alternative for the reconstruction of the hypopharynx, especially in cases where the larynx is preserved.

## Introduction

Reconstruction of the hypopharynx and cervical esophagus poses significant challenges. While several techniques have been described, few reports of the ileal free flap have been published [1], [2], [3], [4]. We present six cases of hypopharyngeal and cervical esophageal reconstruction with larynx preservation using ileal free flap. Our objective is to demonstrate the feasibility of using this technique for reconstructing the hypopharynx and esophagus, considering the anatomical distinctions of the ileum that make it particularly suitable in select cases, especially when the larynx is preserved.

## Surgical technique summary

The surgical procedure began with a median neck incision, extending from the hyoid region to the suprasternal notch, facilitating exposure of the airway. Cervical vessels were meticulously dissected to ensure precision. Special attention was given in cases requiring larynx preservation to prevent any inadvertent damage to the recurrent laryngeal nerves.

For graft harvesting, a median laparotomy was performed, and a segment of the ileum, approximately 20 cm proximal to the ileocecal valve, was identified. The vascular pedicle between the second arches proximal to the surface of the ileum was carefully identified and dissected until reaching a length of 2 cm along its longest axis. The isolated segment was then perfused with a heparin solution, and its proximal end was marked for identification during cervical reconstruction. Restoration of ileal continuity was achieved through an end-to-end anastomosis.

**Figure 1:**
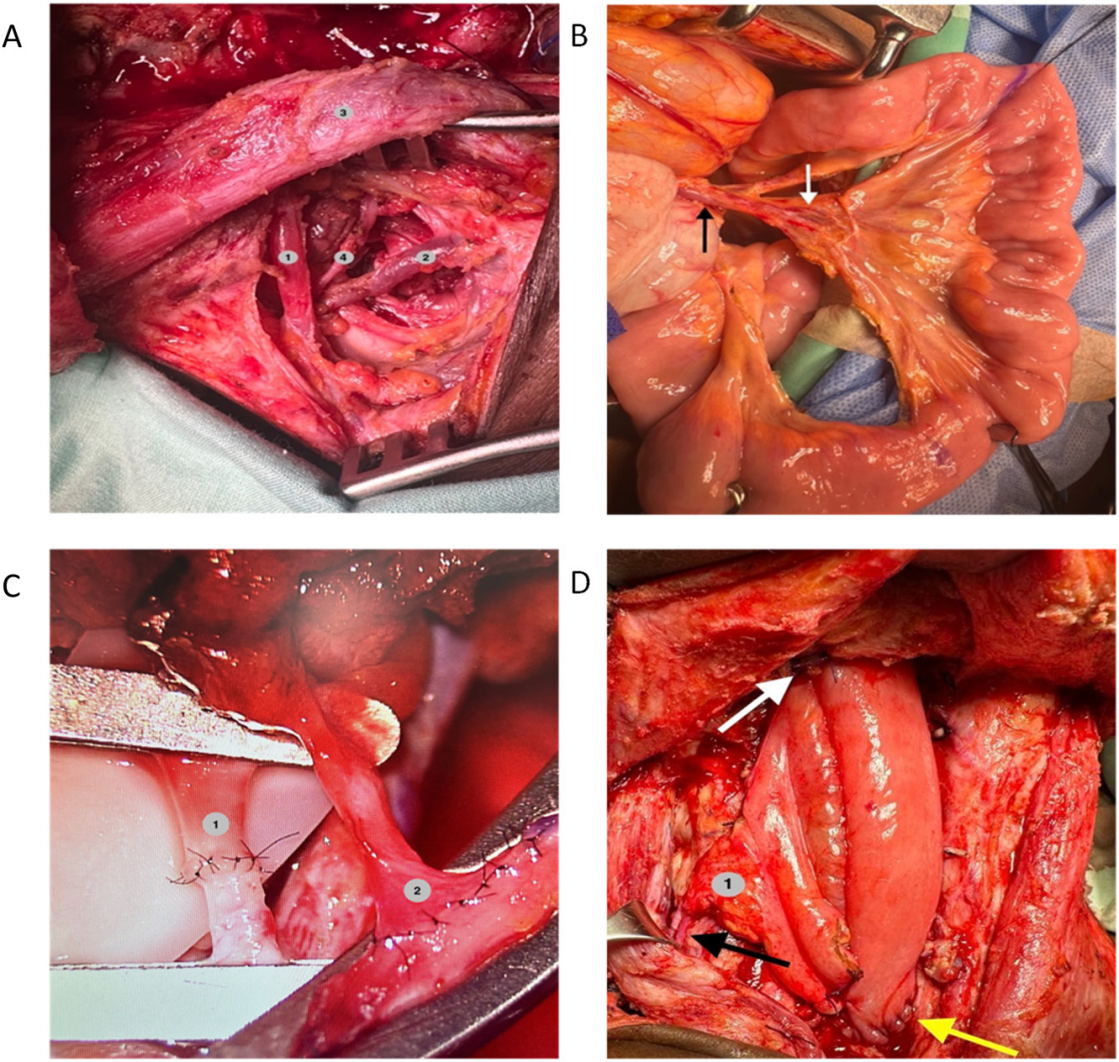
Surgical technique for hypopharynx reconstruction with ileal free flap. (A) Vascular neck dissection. (1) Omohyoid muscle; (2) ascending cervical vein; (3) sternocleidomastoid; (4) transverse cervical artery. (B) Ileal vascular pedicle. Black arrow: ileal vein; white arrow: ileal artery. (C) Microvascular anastomoses. (1) Transverse cervical artery branch-ileal branch anastomoses; (2) ascending cervical vein branch-ileal branch anastomoses. (D) Hypopharynx reconstruction. (1) Omentum; black arrow: vascular anastomoses; white arrow: hypopharyngeal anastomoses; yellow arrow: esophageal anastomoses.

**Figure 2:**
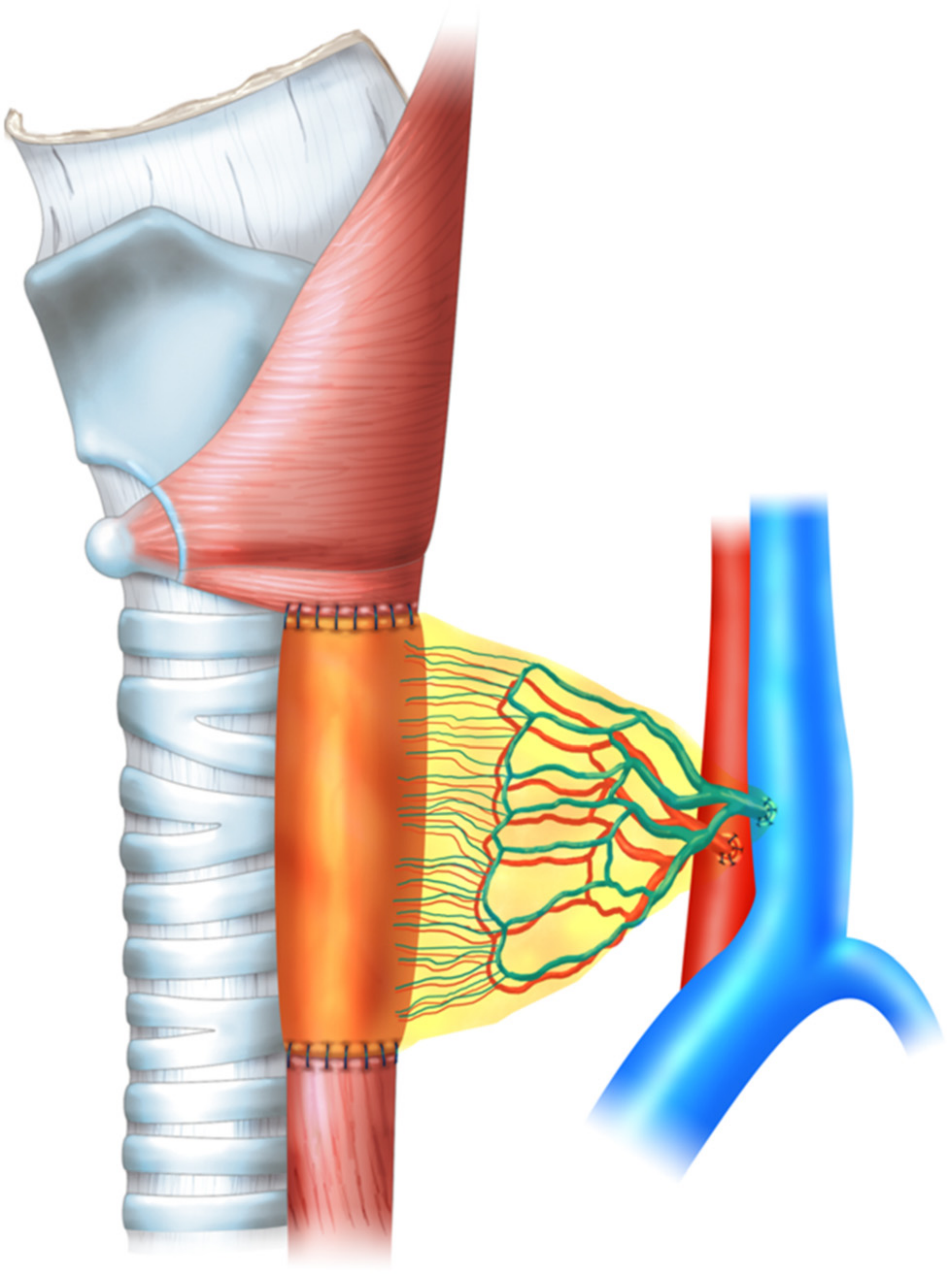
Hypopharyngeal and cervical esophageal reconstruction in larynx-preserved digestive conduit defects.

Subsequently, the proximally marked end of the ileum segment was anastomosed to the hypopharynx in a terminolateral fashion with interrupted sutures using nonabsorbable monofilament, and the distal end was similarly anastomosed to the cervical esophagus, thus reconstructing the digestive conduit defect. The remaining peritoneum was placed to cover all the anastomoses, securing it to the operating field and hemostasis was ensured before closing the incision (Figures 1 and 2).

## Case presentation

Six consecutive cases were reported (age range 17–75; 2 females). Tables 1 and 2 summarize patient characteristics and postoperative outcomes. All patients were diagnosed and treated at a tertiary care university hospital in Cali, Colombia between May 2017 and June 2022.

**Table 1:** Patient characteristics and surgical procedures.

Case	Gender	Age	Past medical history	Indication for surgery	Surgical procedures	Larynx preservation
1	Male	53	– Esophageal perforation following foreign body ingestion	Esophageal stenosis	– Hypopharyngeal and cervical esophagus resection	Yes
					– Reconstruction with ileal free flap	
2	Female	17	– Corrosive injury following caustic ingestion	Esophagogastric stenosis	– Esophagogastric resection	Yes
					– Reconstruction with ileal free flap and colon interposition	
3	Male	60	– Esophageal stenosis following radiotherapy for esophageal cancer	Jejunal free flap failure	– Jejunal free flap resection	Yes
			– Tumor excision and reconstruction with jejunal free flap		– Reconstruction with ileal free flap	
4	Male	75	– Tumor excision of an invasive laryngeal cancer and reconstruction with jejunal free flap	Iatrogenic jejunal free flap stenosis	– Jejunal free flap resection	No
					– Reconstruction with ileal free flap	
5	Male	54	– Tumor excision of an invasive laryngeal cancer	Tumor excision	– Hypopharyngeal and cervical esophageal resection	No
					– Reconstruction with ileal free flap	
6	Female	73	– Esophageal stenosis following radiotherapy for esophageal cancer	Esophageal stenosis	– Hypoharyngeal and cervical esophagus resection	Yes
					– Reconstruction with ileal free flap	

**Table 2:** Postoperative and swallowing outcomes.

Case	Esophagoscopy (24–48 h)	Esophagoscopy (10–14 days)	Esophagoscopy (3 months)	Day pasty diet began	Day solid diet began	Videofluoroscopic swallowing study (20–80 days)	Outpatient follow-up (month 1)	Outpatient follow-up (month 6)
1	– Vital 100 %	– Vital 100 %	– Vital 100 %	15	20	Normal	Diet without restrictions	Diet without restrictions
	– No stenosis	– No stenosis	– No stenosis					
2	– Vital 100 %	– Vital 100 %	– Vital 100 %	20	25	Normal	Diet without restrictions	Diet without restrictions
	– No stenosis	– No stenosis	– No stenosis					
3	– Vital 100 %	– Vital 100 %	– Vital 100 %		No	Partial obstruction distal to the ileal free flap (tumor recurrence)	Tolerating liquid diet	Tube feeding due to complete obstruction (tumor recurrence)
	– No stenosis	– No stenosis	– No stenosis					
4	– Vital 100 %	– Vital 100 % – Proximal and distal anastomoses edema – Proximal stenosis 40 %	– Vital 100 %	15	No	Dysphagia (solids)	Tolerating soft solid diet	Tolerating soft solid diet
	– Proximal and distal anastomoses edema		– No stenosis					
5	– Vital 100 %	– Vital 100 %	– Vital 100 %	14	20	Normal	Diet without restrictions	Diet without restrictions
	– No stenosis	– No stenosis	– No stenosis					
6	– Vital 100 %	– Vital 100 %	– Vital 100 %	81	88	Dysphagia (pasty)	Tube feeding due to dysphagia (liquids)	Diet without restrictions
	– No stenosis	– No stenosis	– No stenosis					

Indications for surgery were tumor excision, post tumor excision jejunal free flap graft failure, postradiation esophageal stenosis, corrosive injury due to caustic ingestion, and cervical esophageal perforation due to foreign body ingestion. The larynx was preserved in four cases.

**Figure 3:**
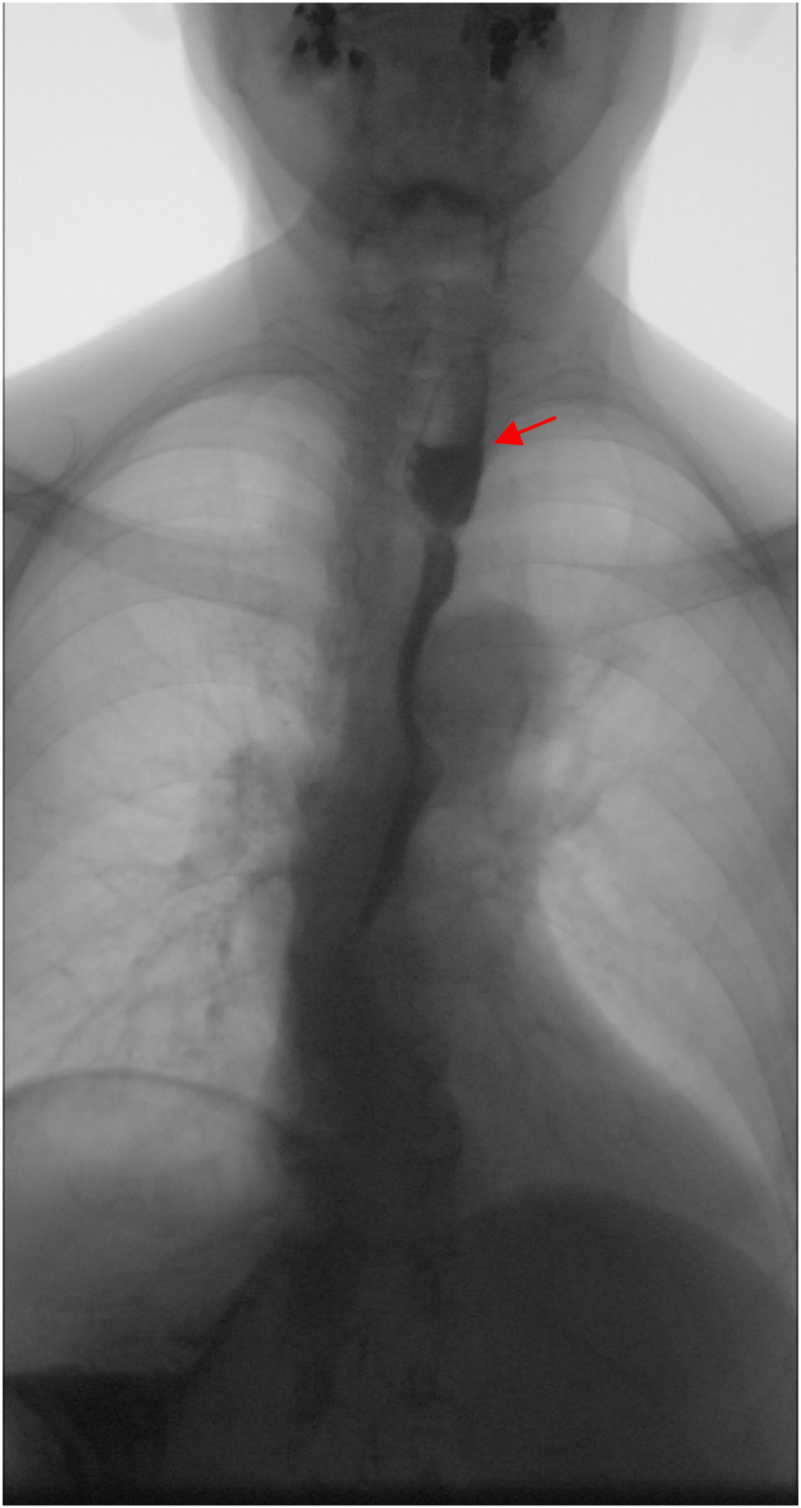
Videofluoroscopic swallowing study at day 72 after hypopharyngeal and cervical esophageal reconstruction. The red arrow points to the patent ileal free flap segment.

Postoperative outcomes include (1) graft survival as evidenced on esophagoscopies at postoperative 24–48 h and 10–14 days, (2) onset of swallowing as evidenced by the first successful intake of a pasty diet meal, (3) digestive conduit functioning as evidenced by videofluoroscopic swallowing tests (Figure 3) at postoperative 20–80 days, and (4) the state of deglutition as reported by the patients at first- and sixth-month outpatient follow-ups.

Graft survival rate was 100 % and videofluoroscopic swallowing studies at postoperative day 20–80 were normal in three cases. Two abnormal cases presented with stenosis: one at the distal ileo-esophageal anastomosis and the other involving both esophageal anastomotic sites. Both responded well to endoscopic dilations. The third case involved tumor recurrence, causing extrinsic compression of the distal esophagus, requiring tube feeding due to obstruction. The latter patient expired due to tumor recurrence and subsequent malignant hypercalcemia.

The patients initiated oral intake 48 h postoperatively, without experiencing subsequent complications such as wound dehiscence, fistula formation, or peritonitis. At the 3-month follow-up assessment, all grafts demonstrated a 100 % survival rate, with no associated complications.

## Discussion

Several reconstruction techniques have been described, including locoregional tubularized island flaps, gastric pull-up, and jejunum free flaps [5], [6], [7], [8]. Our experience suggests that the ileum presents advantages over other tissues for hypopharyngeal reconstruction and larynx preservation. This innovative approach simplifies the surgical process and enhances postoperative outcomes.

While the technique of free jejunum grafting and the radial fasciocutaneos flap have been more extensively documented, advances in microsurgery have enabled the feasibility of hypopharyngeal and cervical esophageal reconstructions using the ileal free flap [3]. In comparison with the radial fasciocutaneous flap, the ileum exhibits peristalsis, facilitating postoperative swallowing recovery. Furthermore, it requires only the anastomosis of its edges, in contrast to the radial flap which requires a third anastomosis [9].

Additionally, the ileum outperforms the jejunum due to its thinner intestinal walls and a diameter similar to that of the cervical esophagus, facilitating a more feasible anastomosis with the walls of the hypopharynx [10]. Furthermore, the dimensions of the ileum make it more technically suitable for accommodating restricted spaces, as notably observed in four of our cases where the larynx was preserved.

No focal necrosis or anastomotic leakages were observed in any of the esophagoscopies. Moreover, 80 % of our patients maintained oral feeding 6 months postoperatively, while the remaining patient required tube feeding due to malignancy recurrence causing extrinsic esophageal obstruction.

The primary limitation of this study is its lack of comparison with another technique, which precludes a definitive assessment of the superiority of the ileal free flap over other tissues. Nevertheless, our findings demonstrate that the ileal free flap serves as an innovative surgical alternative to address complex anatomical challenges in the reconstruction of the hypopharynx and cervical esophagus, particularly in cases where laryngeal preservation is crucial. This is attributed to its anatomical compatibility, facilitating digestive anastomosis with the hypopharynx and leading to favorable clinical outcomes.

## Conclusions

The ileal free flap represents a promising and innovative alternative for hypopharyngeal and cervical esophageal reconstruction, particularly in cases where there is limited cervical space, such as when larynx preservation is required. Our study highlights its potential as a technically feasible and successful approach in addressing complex anatomical challenges.
